# Case Report: Focused shockwave therapy (fESWT) in thumb carpometacarpal joint osteoarthritis: a single case study

**DOI:** 10.3389/fresc.2025.1716305

**Published:** 2026-01-12

**Authors:** Timmy Gustafsson, Sofia Ryman Augustsson

**Affiliations:** 1 Unit of Physiotherapy, Department of Health, Medicine and Caring Sciences, Linköping University, Linköping, Sweden; 2 Department of Sport Science, Linnaeus University, Kalmar, Sweden

**Keywords:** CMC joint, grip strength, ICD-10 M.18, musculoskeletal pain, physiotherapy, QuickDASH

## Abstract

**Background:**

Several studies have demonstrated the efficacy of shock wave therapy in managing OA-related pain and improving joint function but evidence specific to thumb CMC joint OA remains limited. Therefore, the aim of this study was to evaluate the effectiveness of fESWT in reducing pain and improving function as well as to assess the safety and tolerability of this intervention, providing insight into its potential role as a non-invasive and long-term alternative for patients unresponsive to conventional therapies or seeking to avoid surgery.

**Methods:**

A 64-year-old woman with radiographically confirmed bilateral CMC OA and persistent symptoms unresponsive to prior conservative treatments received three weekly fESWT sessions. Energy flux density was individually adjusted per session based on tolerance. Outcome measures included the QuickDASH questionnaire and grip strength testing, assessed at baseline, 2-, 8-, 26-, and 52-weeks post-intervention.

**Results:**

Treatment was well tolerated with no adverse events. QuickDASH scores improved from 20.5 at baseline to 2.3 at 52 weeks, indicating an improvement in pain and functional disability. Grip strength increased by 29.5% in the right hand and 17.4% in the left hand over the same period. Subjectively, the patient reported pain relief, functional improvement, and sustained benefit at one-year follow-up.

**Conclusions:**

This case demonstrates the potential of individualized fESWT as a safe and effective intervention for thumb CMC OA, with improvements in pain and function lasting up to 12 months. These findings support further investigation of fESWT in larger controlled studies and highlight the importance of individualized dosing strategies in clinical practice.

## 1 Introduction

Thumb Carpometacarpal (CMC) joint osteoarthritis (OA) is a common degenerative condition affecting the base of the thumb (1, 2). Thumb CMC joint is a saddle-shaped joint allowing for a wide range of thumb movements (3). OA primarily involves the first CMC joint and is prevalent among middle-aged and elderly populations, particularly affecting women (1, 4). OA in this joint results from the progressive deterioration of articular cartilage, changes in subchondral bone, and synovial inflammation (3, 5). Mechanical stress, genetic predisposition, hormonal factors, and previous joint injuries are contributing factors (3, 5). The degradation of cartilage leads to joint space narrowing, osteophyte formation, and altered joint mechanics, causing pain and functional impairment (3, 5). Thus, the condition is characterized by pain, swelling, stiffness, and reduced function, significantly impacting daily activities and overall quality of life (1, 2, 5). Patients typically present with pain at the base of the thumb, often exacerbated by pinching or gripping activities (5). Physical examination may reveal tenderness, swelling, and a positive grind test. Radiographic findings, including joint space narrowing, subchondral sclerosis, and osteophytes, confirm the diagnosis (5). Magnetic resonance imaging (MRI) can provide detailed visualization of cartilage loss and soft tissue changes (5).

Surgical treatment for thumb CMC joint OA can be effective in advanced cases but is associated with considerable costs, risks, and recovery time (6). An economic analysis estimated the average cost of CMC arthroplasty in the U.S. to exceed $10,000 per case, not accounting for indirect costs such as time off work and rehabilitation (7). In contrast, conservative management, including splinting, manual therapy, and physical modalities, poses significantly lower direct costs and fewer systemic risks (8). Surgical interventions are typically reserved as a last resort, with non-operative management preferred as the initial approach; consequently, multimodal conservative strategies are recommended as the first-line treatment (6). Non-surgical options may include non-steroidal anti-inflammatory drugs, education, therapeutic exercises, corticosteroid or hyaluronic injections, physiotherapy modalities, manual therapy (6, 9), and orthotic devices (6, 9, 10). Systematic reviews have shown that combining therapeutic exercises with manual therapy and orthotic interventions can effectively improve pain and function in the short term for patients with thumb CMC joint OA (9, 11).

One conservative treatment option is focused extracorporeal shockwave therapy (fESWT), a non-invasive modality that has gained increasing attention for its potential benefits in musculoskeletal disorders, including OA (12). This fESWT delivers high-energy acoustic waves to targeted tissues, inducing mechanical and biological effects that promote healing and pain relief. The mechanism of action includes (12): 1) Mechanotransduction: Shockwaves generate mechanical stress, stimulating cellular responses and tissue regeneration; 2) Pain Modulation: Shockwaves may inhibit nociceptors and modulate pain signalling pathways; 3) Angiogenesis: ESWT promotes the formation of new blood vessels, enhancing tissue perfusion and repair; 4) Anti-inflammatory Effects: Reduction in local inflammatory mediators and improvement in local tissue environment.

Several studies have demonstrated the efficacy of fESWT in managing OA-related pain and improving joint function (13). For instance, studies on knee OA have shown significant pain reduction and functional improvement following fESWT (14). The positive outcomes are attributed to the anti-inflammatory effects, cartilage repair stimulation, and enhanced microcirculation induced by shockwaves (15). While most evidence relates to larger joints such as the knee and shoulder, research specific to thumb CMC OA remains limited. Nonetheless, two studies have yielded promising findings (16, 17), underscoring the need for continued investigation. In the study by Covelli et al. (16), shock wave therapy demonstrated superior improvements in pain and functional outcomes over time compared with exercise therapy. However, the study assessed only subjective measures of function, without incorporating objective evaluations such as strength testing. In addition, the study has also been criticized mainly due to its use of an inappropriate statistical method that fails to adequately compare the two treatment modalities (18). In the study by Ioppolo et al. (17), shock wave therapy was compared with hyaluronic acid injections and was found to provide greater long-term pain relief, improved pinch strength, and reduced hand disability in patients with first carpometacarpal joint osteoarthritis. Both studies demonstrated positive treatment effects at the 6-month follow-up. However, to our knowledge, no previous research has reported outcomes of fESWT for thumb CMC OA beyond this time point. This case provides insight into the potential long-term persistence of treatment effects, which could guide the rationale and design of future randomized controlled trials.

Given the promising results of fESWT in other joints and the limited treatment options for thumb CMC joint OA, this single case study primarily aimed to evaluate the effectiveness of fESWT in reducing pain and improving function. A secondary aim was to assess the safety and tolerability of this intervention, providing insight into its potential role as a non-invasive and long-term alternative for patients unresponsive to conventional therapies or seeking to avoid surgery.

## 2 Methods

### 2.1 Study design and procedure

This single-case study was conducted at a physiotherapy clinic specializing in orthopaedic and sports medicine in southeastern Sweden. The aim was to evaluate the therapeutic effects of focused extracorporeal shockwave therapy (fESWT) on a patient with persistent symptoms.

A baseline assessment was performed prior to the intervention and included patient reported outcome measure (PROM) and strength assessments. This served as the reference point for subsequent evaluations. Immediately following the baseline assessment, the first fESWT session was administered. The second treatment session was conducted one week after baseline. During week 2, a follow-up assessment was performed, including measurements of strength and PROM. This was followed by the third and final fESWT session. To monitor treatment effects over time, follow-up assessments were conducted at 8-, 26-, and 52-weeks post-baseline, using the same outcome measures as at baseline. These included both patient-reported and performance-based outcomes to ensure a comprehensive evaluation.

### 2.2 Case presentation

The patient was a 64-year-old woman who presented to the clinic with bilateral thumb pain, more pronounced on the right side. The selection criterion was chronic pain associated with CMC OA that had not responded to previous conservative treatment. She had retired from her work as a dental nurse, primarily due to chronic neck pain, but also because of persistent thumb pain during daily work activities. Radiographs confirmed bilateral thumb CMC OA (ICD-10 M.18), classified as Eaton grade 2, indicating decreased joint space and osteophyte formation (19). More than ten years prior, she had undergone bilateral carpal tunnel release surgery with good outcomes. The patient reported daily pain both during activities and at rest, with resting pain described as particularly difficult to manage. She experienced stabbing pain during activity, difficulty gripping, and trouble carrying objects on a daily basis. She had previously tried various treatments, including three different orthoses and physical therapy interventions such as stretching and exercise, but experienced only minor improvements. She had occasionally used non-steroidal anti-inflammatory drugs (NSAIDs) when necessary, which had negligible effect, and did not receive any injections. She engaged in general gym training two times per week throughout the period before, during, and after the intervention. However, she did not perform any specific rehabilitation exercises targeting the hand or fingers. Prior to the intervention, the patient was informed about the potential benefits of fESWT, the underlying mechanisms of action, the number of planned treatment sessions, and what to expect following each session. The study was performed in accordance with the declaration of Helsinki (20) and informed consent was obtained from the patient for participation in this case report.

### 2.3 Intervention

Shockwave therapy was applied using a fESWT device (Intelect FSW, Chattanooga, Enovis) at the most painful spots (identified during treatment with the handpiece of the shockwave device) at the thumb CMC joint from both the palmar and volar aspect of view. The patient was seated with the forearm supinated to expose the first CMC joint. The shockwave applicator was positioned perpendicular to the skin, with minor adjustments as needed to maintain full coupling. Ultrasound gel ensured effective energy transmission, and the applicator was held in stable contact without excessive pressure or movement. The intervention consisted of three shockwave therapy sessions, administered at one-week intervals:

Session 1: 2,000 shockwaves (SW), 4 Hertz (Hz), 0,07 mJ/mm^2^
Session 2: 2,000 SW, 4 Hz, 0,10 mJ/mm^2^
Session 3: 2,000 SW, 4 Hz, 0,12 mJ/mm^2^

The treatment protocol was adjusted gradually in terms of energy level, moving from 0.07 mJ/mm^2^ to 0.12 mJ/mm^2^ by the third session.

No formal NSAID washout period was implemented. However, the patient was instructed to refrain from NSAID use for 3–4 days before and after each ESWT session to avoid potential interference with shockwave-induced inflammatory and regenerative processes. During the intervention and the 52-week follow-up, no additional treatments were received (i.e., no orthoses, specific exercises or pain medication over time).

### 2.4 Outcome measures

Pain intensity was measured at each session using Visual Analog Scale for pain (VAS) (21, 22). Patient-reported upper limb disability and symptoms were assessed using the Quick Disabilities of the Arm, Shoulder and Hand (QuickDASH) questionnaire (23). The instrument consists of 11 items that measure physical function and symptoms related to arm, shoulder, and hand conditions. Each item is rated on a 5-point Likert scale, and the total score is transformed to a scale from 0 (no disability) to 100 (severe disability) (24). The QuickDASH has been validated for use in musculoskeletal disorders of the upper extremity and demonstrates good reliability, validity, and responsiveness in both clinical and research settings (23, 25). In addition to patient-reported outcomes, grip strength was measured using a Jamar® handheld dynamometer. Measurements were taken in the standard position (seated, elbow flexed at 90 °, forearm in neutral position) (26), and the average of three trials was recorded for each hand at baseline, one week post-intervention, and at 8-, 26-, and 52-week follow-ups.

### 2.5 Data analysis

Data analysis was performed through both visual inspection of graphed data and statistical procedures. Statistical analyses included individual-level effect size estimation (ES), expressed as the standardized mean difference (Cohen's d) (27) for grip-strength and QuickDASH, and autoregressive integrated moving average (ARIMA) models for QuickDASH. The minimal important change was defined as 10.3 points for the QuickDASH (28) and 0.84 kg for grip strength (29). Calculations were performed using a scientific calculator (Timing, CH-257, China) and IBM SPSS Statistics (Version 30.0; IBM Corp., Armonk, NY, USA).

## 3 Results

**Figure 1:**
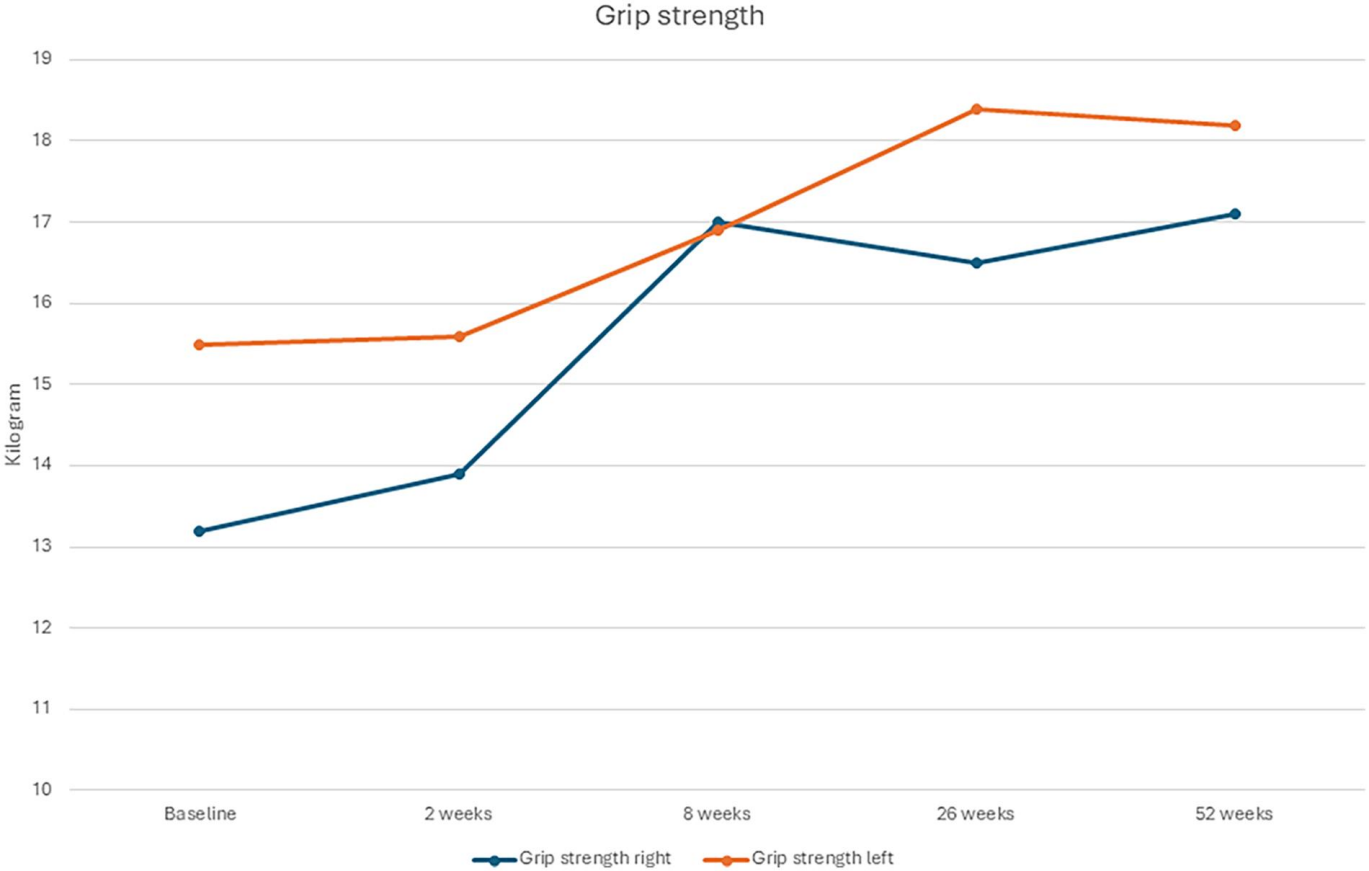
Grip-strength from baseline to 52 weeks of follow-up.

**Figure 2:**
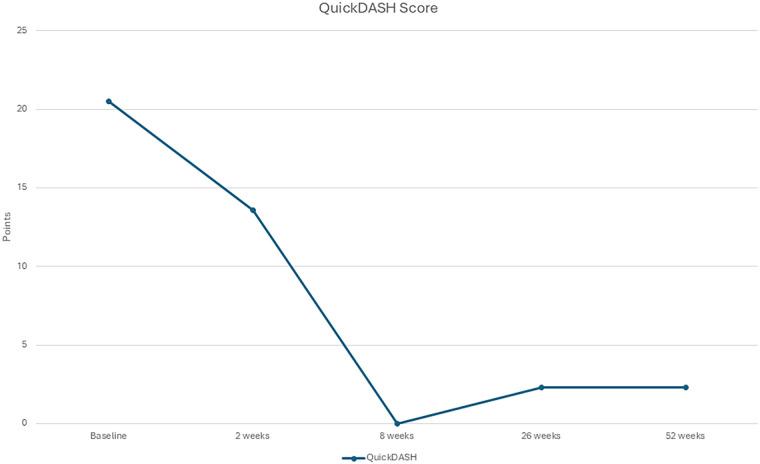
QuickDASH from baseline to 52 weeks of follow-up.

**Figure 3:**
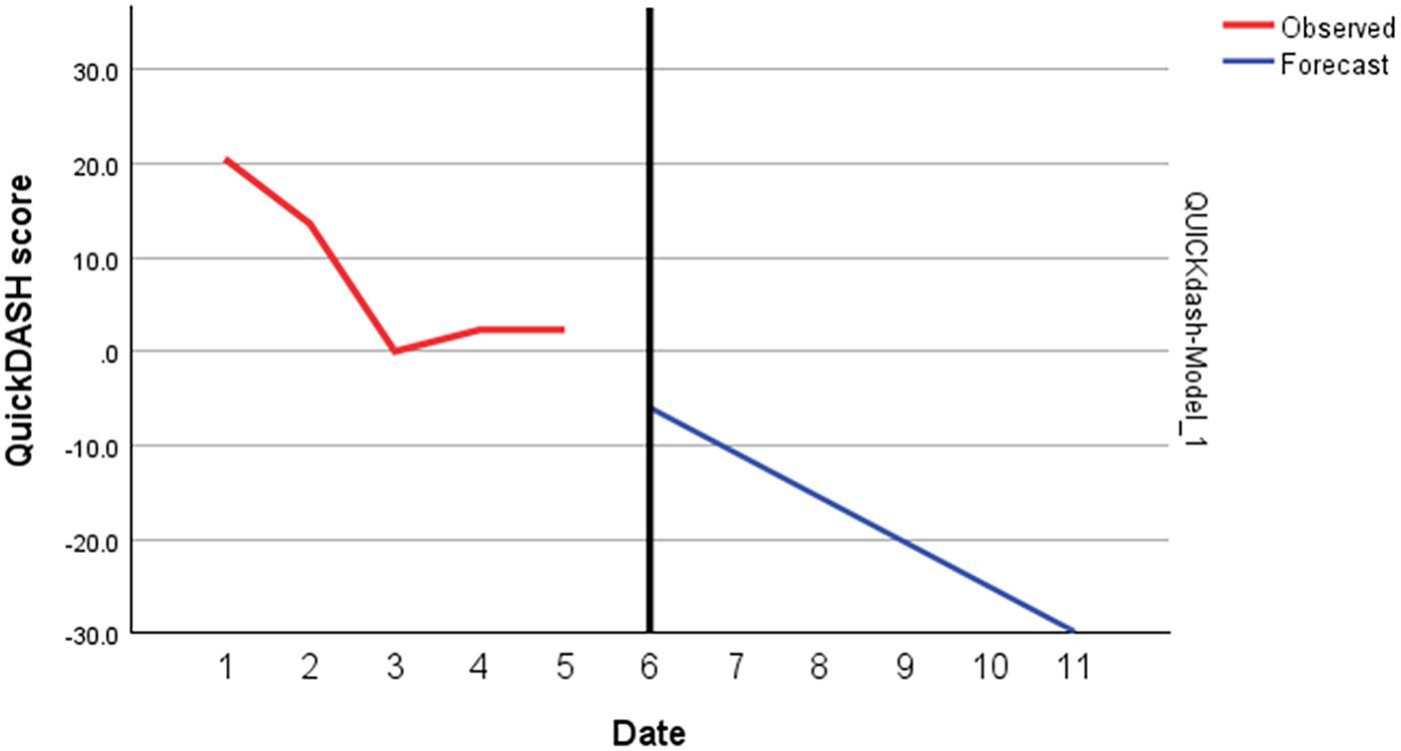
ARIMA model for QuickDash.

The patient tolerated all three fESWT sessions well, initially reporting moderate discomfort (VAS 6–7), which decreased over time (VAS 5), with no local reactions such as redness or bruising. Subjectively, the patient reported a marked reduction in pain and improvements in functional abilities, including gripping, pinching, and carrying, which were reflected in the outcome measures. In between the first and second session the patient was pain free in the left thumb for three days and had a decrease of pain in the right thumb. Regarding the week between the second and third sessions the patient was pain free for a week in the left thumb and had additionally decreased pain in the right thumb. Right hand improved in strength from 13.2 kg to 17.1 kg (+29.5%; ES = 1.9; Stationary *R*^2^ = 0.81), and the left hand improved from 15.5 kg to 18.2 kg (+17.4%; ES = 1.4; Stationary *R*^2^ = 0.80) after a year follow-up (Figure 1 and Table 1). A reduction in the total QuickDASH score was observed, indicating an improvement in pain and functional disability (ES = 2.6; Stationary *R*^2^ = 0.59, Figure 2, 3). The improvements observed in QuickDASH and grip strength exceeded the minimal clinically important difference threshold for thumb CMC OA (28, 29). No lifestyle changes were reported during follow-up; the patient maintained a structured retired life.

**Table 1:** QuickDASH scores and grip strength from baseline to 52 weeks follow-up.

Timepoint	QuickDASH score	Grip strength, right/left
Baseline	20.5	13.2/15.5 kg
2 weeks	13.6	13.9/15.6 kg
8 weeks	0.0	17.0/16.9 kg
26 weeks	2.3	16.5/18.4 kg
52 weeks	2.3	17.1/18.2 kg

## 4 Discussion

This case highlights the promising role of focused extracorporeal shockwave therapy (fESWT) in treating thumb CMC OA. The patient experienced rapid and clinically meaningful reductions in pain and disability, confirmed both subjectively and through QuickDASH scores, with functional gains sustained at one-year follow-up. These findings align with growing evidence that fESWT can be effective in managing musculoskeletal disorders, including OA of larger joints such as the knee and shoulder (30, 31).

The patient reported marked improvements in grip, pinch, and carrying tasks, with complete pain relief in the left thumb and substantial relief in the right after only two sessions. Grip strength also improved, consistent with reports linking fESWT to functional recovery (28). Although grip strength is influenced by multiple factors, such as pain, OA severity, comorbidities, sex, BMI, and hand dominance (32, 33), the observed improvement corresponded well with the patient's subjective reports. No adverse events were recorded.

The unique anatomy and functional demands of the thumb joint necessitate tailored therapy. Unlike weight-bearing joints, the thumb is critical for fine motor control, requiring precision in treatment application. In this case, energy flux density was adjusted session by session according to pain tolerance, a strategy supported by prior studies showing dose-dependent and individualized responsiveness in ESWT (34–36). Standardized fixed protocols (17) may overlook patient variability, such as joint alignment, soft tissue condition, pain threshold, and disease stage, underscoring the need for individualized dosing strategies.

Despite encouraging results, limitations should be acknowledged. Imaging was not used to quantify tissue-level changes, limiting mechanistic interpretation. Additionally, while prior studies suggest ESWT effects may plateau without maintenance therapy (14), this case demonstrated durable improvement, warranting further investigation. There is also a potential risk of recall bias during follow-up assessments, particularly for measures with subjective components such as patient-reported outcome measures (PROMs). To minimize this limitation, both subjective (PROMs) and objective (grip strength) assessments were included. Furthermore, case studies are inherently susceptible to reporting bias, as positive outcomes are more likely to be published. Therefore, future research should also report neutral or negative findings to provide a more comprehensive understanding of the effects of focused extracorporeal shock wave therapy (fESWT) in patients with thumb CMC OA. From a health economics perspective, fESWT offers an attractive alternative to surgery. It is non-invasive, outpatient-based, and does not typically require rehabilitation or time off work. Although device and training costs are notable, repeated sessions remain far less expensive than surgical intervention (37). If fESWT can delay or prevent surgery in select patients, the potential savings could be substantial (38).

Lastly, given the single-case design, the results are not generalizable, and without a control group, it is difficult to rule out placebo effects or confounding factors (e.g., lifestyle or activity changes). Therefore, the findings from the present study should be considered hypotheses for future randomized controlled trials. Still, this case suggests that individualized fESWT protocols, guided by patient feedback, clinical progression, and functional goals, may provide superior outcomes compared to standardized regimens. Given its favorable safety profile and cost-effectiveness, fESWT warrants further exploration as part of multimodal management strategies for thumb CMC OA in larger controlled trials.

## 5 Conclusion

This case supports the potential role of individualized, adjusted fESWT as a safe and effective non-invasive treatment for thumb CMC OA. Sustained improvements in pain, grip strength, and function were observed up to 52 weeks, suggesting durable effects beyond short-term symptom relief. While these findings cannot be generalized from a single patient, they highlight the need for controlled studies investigating personalized ESWT protocols in thumb OA, with attention to both clinical outcomes and cost-effectiveness. Findings from the present study may serve as a preliminary step toward the design of future controlled studies evaluating the efficacy of fESWT in thumb CMC OA.

## Data Availability

The raw data supporting the conclusions of this article will be made available by the authors, without undue reservation.
